# Multidisziplinarität als Schlüssel zum Erfolg: Zusammenarbeit zwischen Dermatologie und Rheumatologie zur Optimierung der Patientenversorgung

**DOI:** 10.1007/s00105-022-05051-z

**Published:** 2022-09-07

**Authors:** Petra Staubach, Katrin Veelen, Sebastian Zimmer, Anna Sohn, Berenice M. Lang, Adriane Peveling-Oberhag, Stephan Grabbe, Wiebke Kaluza-Schilling, Andreas Schwarting, Joanna Wegner

**Affiliations:** 1 Hautklinik und Poliklinik, Universitätsmedizin Mainz, Langenbeckstr. 1, 55131 Mainz, Deutschland; 2 Rheumatologie, Universitätsmedizin Mainz, Mainz, Deutschland; 3 Universitäres Centrum für Autoimmunität (UCA) Mainz, Universitätsmedizin Mainz, Mainz, Deutschland

**Keywords:** Autoimmunerkrankungen, Autoinflammation, Autoimmunität, Psoriasisarthritis, Rheumaboard, Autoimmune diseases, Autoinflammation, Autoimmunity, Psoriatic arthritis, Rheuma board

## Abstract

**Hintergrund:**

Patienten mit systemischen Autoimmun- und/oder autoinflammatorischen Erkrankungen (AI/AInf) bedürfen in der Regel einer multidisziplinären Zusammenarbeit durch verschiedene Fachrichtungen.

**Ziel der Arbeit (Fragestellung):**

Wir evaluierten, ob die Etablierung eines multidisziplinären Boards (sog. Rheumaboard [RB]) zur Optimierung der Versorgung von Patienten mit Psoriasisarthritis (PsA) oder anderen AI/AInf führt.

**Material und Methoden:**

Es wurden *n* = 272 Patienten mit AI/AInf eingeschlossen, die in 3 Gruppen eingeteilt wurden; Gruppe 1: 41 Patienten mit oder mit Verdacht auf (V. a.) PsA, von der Dermatologie in der Rheumatologie konsiliarisch avisiert; Gruppe 2: 166 Patienten mit oder mit V. a. PsA, vorstellig in der Dermatologie und im RB; Gruppe 3: 65 Patienten mit anderen AI/AInf, vorstellig in der Dermatologie und im RB. Evaluiert wurde die durchschnittliche Zeit von der initialen Vorstellung bis zur Therapieeinleitung nach erfolgter Beurteilung und Diagnostik durch beide Fachrichtungen. Darüber hinaus wurden die Diagnosesicherung/-bestätigung und die Therapieweiterführung/-optimierung bei allen 3 Gruppen analysiert.

**Ergebnisse:**

Die durchschnittliche Zeitspanne von der initialen Vorstellung bis zur Therapieeinleitung betrug in Gruppe 1 85 ± 42,24 (5 bis 173) Tage, in Gruppe 2 15 ± 13,09 (0 bis 78) Tage und in Gruppe 3 20 ± 16,71 (1 bis 75) Tage. In Gruppe 2 und 3 konnte die Diagnose schneller gesichert oder bestätigt sowie die Wartezeit auf Diagnostik und Therapie deutlich reduziert werden.

**Diskussion:**

Durch die Etablierung eines RB zeigt sich eine signifikante Verkürzung der Zeitspanne zwischen Erstvorstellung und Therapieeinleitung und damit eine deutliche Verbesserung des Versorgungsmanagements bei Patienten mit AI/AInf inklusive Diagnosesicherung und Therapieoptimierung.

**Graphic abstract:**

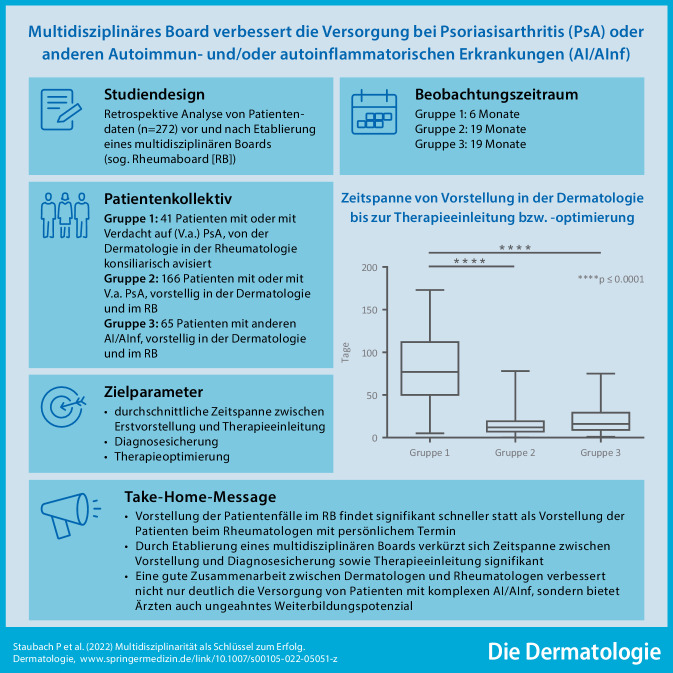

## Abstract (en)

**Background:**

Treatment of patients with systemic autoimmune and/or autoinflammatory diseases (AI/AInf) often requires multidisciplinary collaboration of various medical specialties.

**Objectives:**

We evaluated whether the establishment of a multidisciplinary board, which we termed rheuma board (RB), can contribute to optimization of care for patients with psoriatic arthritis (PsA) or other AI/AInf.

**Materials and methods:**

A total of 272 patients were included in the study. Patients were divided into three groups—group 1: 41 patients with or with suspected PsA, initially assessed in the dermatology department and afterwards presented for consultation in the rheumatology department; group 2: 166 patients with or with suspected PsA presenting in the dermatology department and afterwards discussed by RB; group 3: 65 patients with other AI/AInf presenting in the dermatology department and afterwards discussed by RB. We evaluated the average duration from initial presentation to therapy initiation after completing evaluation and diagnostics by both specialties. In addition, diagnosis confirmation/verification and therapy continuation/optimization were analyzed for all three groups.

**Results:**

The average duration from initial presentation until therapy initiation was 85 ± 42.24 (5–173) days in group 1, 15 ± 13.09 (0–78) days in group 2, and 20 ± 16.71 (1–75) days in group 3. In addition, in groups 2 and 3 confirmation of diagnosis was faster and waiting time for diagnosis and therapy initiation was significantly reduced.

**Conclusions:**

Establishment of a RB results in a significant reduction in the time duration between first presentation and initiation of therapy, and an improvement of care for patients with AI/AInf including confirmation of diagnosis and therapy optimization.

## Hintergrund und Fragestellung

Autoimmun- und autoinflammatorische Erkrankungen (AI/AInf) sind chronisch entzündliche Erkrankungen, die häufig eine Beteiligung mehrerer Organsysteme aufweisen und nicht zuletzt durch die Gefahr der Komorbidität eine interdisziplinäre medizinische Versorgung benötigen. Im Bereich der Dermatologie sind die Rheumatologen hier, neben anderen Disziplinen wie Gastroenterologen, Neurologen, Augenärzten, Radiologen und Kinderärzten, wichtige Kooperationspartner. Um Erstdiagnose, Progredienz der Erkrankung und Komorbidität zu vermeiden oder systemische Therapie einzuleiten/zu optimieren ist ein professionelles und zeitnahes Patientenmanagement gefordert.

Aufgrund der systemischen Erkrankung benötigen Patienten mit AI/AInf zur Optimierung der Diagnostik und Therapie eine fachübergreifende Betreuung, die auch die potenzielle Komorbidität berücksichtigt. In der Dermatologie ist v. a. die Diagnose Psoriasisarthritis (PsA), die seit 2019 wie die Psoriasis von der Weltgesundheitsorganisation als Systemerkrankung anerkannt ist [20], die häufigste Erkrankung in diesem Gebiet. PsA ist eine chronisch entzündliche Arthritis, die mit Psoriasis einhergeht. Die Epidemiologie der PsA ist heterogen und weist große Unterschiede zwischen den verschiedenen Bevölkerungsgruppen auf. Schätzungen zufolge liegt die Prävalenz der PsA in der Allgemeinbevölkerung bei 0,05–0,25 % und bei Psoriasispatienten bei 6–41 % [2, 14]. Diese Variabilität der PsA bei Psoriasis ist teilweise auf die mangelnde Aufmerksamkeit beginnender Symptome und damit eine fehlende Diagnose zurückzuführen. Patienten mit Psoriasis sind oft von noch weiterer Komorbidität betroffen, insbesondere dem metabolischen Syndrom [16], Depressionen [10], chronisch entzündlichen Darmerkrankungen [12] oder Uveitis [5]. Die Psoriasis per se gilt auch als ungünstiger kardiovaskulärer Risikofaktor [8, 11, 13, 17], was zu einer Verkürzung der individuellen Lebenserwartung um 3 bis 4 Jahre führt [9]. Die Lebensqualität der Psoriasispatienten ist nicht zuletzt durch Juckreiz, Schmerzen und Stigmatisierung erheblich eingeschränkt [4]. Andere häufig vorkommenden AI (z. B. systemischer Lupus erythematodes und systemische Sklerose) gehören zu den häufigsten Todesursachen bei Frauen im jungen und mittleren Alter in den Vereinigten Staaten [19]. Die Inzidenzraten der einzelnen AI variieren [6]. Sie gehören zu den komplexen, potenziell lebensbedrohlichen Systemerkrankungen, die – bei verzögertem oder unzureichendem Management – zu schweren bleibenden Schäden an praktisch allen betroffenen Geweben und Organen führen können.

Zur Optimierung der Patientenversorgung in diesen Kohorten etablierten die Hautklinik und die Rheumatologie der Universitätsmedizin Mainz im Rahmen des Universitären Centrums für Autoimmunität (UCA) Mainz im September 2015 ein multidisziplinäres Board (sog. Rheumaboard [RB]). Bekannt sind interdisziplinäre Boards bereits in verschiedenen Fachrichtungen v. a. in der Onkologie in Form von Tumorboards. Es optimiert das Versorgungsmanagement und somit schnellere Diagnosesicherung und eine zeitnahe Therapieeinleitung bzw. -umstellung unter Berücksichtigung der Komorbidität [1].

Ziel dieser Arbeit war die Untersuchung, ob die Etablierung eines RB zur Optimierung der Versorgung von Patienten mit PsA, anderen AI oder AInf führt. Zu diesem Zweck wurde neben der Sicherung der Diagnose die durchschnittliche Zeitspanne von der Vorstellung in der dermatologischen Psoriasis- bzw. AI/AInf-Spezialsprechstunde bis zur Vorstellung bei den Rheumatologen (konsiliarisch oder im RB) und somit bis zur Diagnosesicherung/-bestätigung sowie Therapieweiterführung/-optimierung analysiert.

## Studiendesign und Untersuchungsmethoden

**Figure 1:**
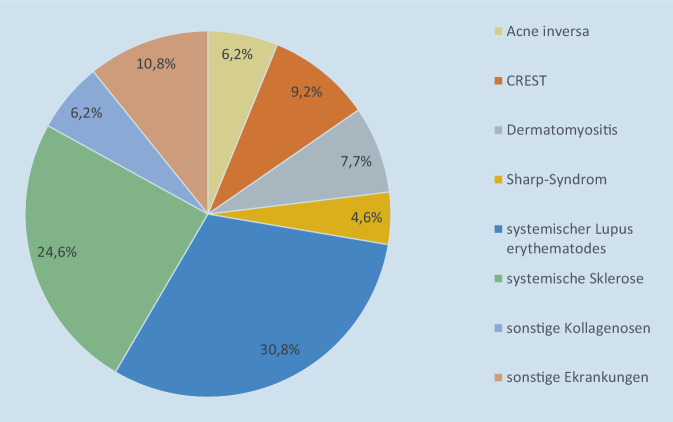

Die Daten von insgesamt *n* = 272 Patienten wurden retrospektiv anhand der elektronischen Patientenakte ausgewertet. Die Erhebung wurde mit der Zustimmung der Ethikkommission der Landesärztekammer Rheinland-Pfalz durchgeführt. Alle Patienten haben sich zunächst in den Spezialsprechstunden der Hautklinik der Universitätsmedizin Mainz vorgestellt. Patienten wurden in 3 Gruppen eingeteilt. In der Gruppe 1 wurden insgesamt 41 Patienten mit gesicherter Diagnose PsA oder mit Verdacht auf (V. a.) PsA im Zeitraum von 6 Monaten (03/2015 bis 08/2015) durch einen Rheumatologen – von der Dermatologie veranlasst – konsiliarisch gesehen, davon wurden 2 Patienten als Notfall in der rheumatologischen Sprechstunde vorstellig. In der Gruppe 2 wurden 166 Patienten mit PsA oder mit V. a. PsA im Zeitraum von 19 Monaten (09/2015 bis 03/2017) im RB von Dermatologen vorgestellt und mit Rheumatologen interdisziplinär im RB besprochen. In der Gruppe 3 wurden 65 Patienten mit anderen AI wie systemischem Lupus erythematodes oder systemischer Sklerose (Abb. 1) im gleichen Zeitraum von 19 Monaten (09/2015 bis 03/2017) im RB besprochen (Tab. 1).

**Table 1:** 

	Gruppe 1	Gruppe 2	Gruppe 3
n *(%) *[∑ = 272]	41 *(15)*	166 *(61)*	65 *(24)*
Untersuchungszeitraum *(Monate)*	03/15–08/15 *(6)*	09/15–03/17 *(19)*	09/15–03/17 *(19)*
Diagnosen	PsA o. V. a. PsA	PsA o. V. a. PsA	Andere AI^a,b^
Rheumatologische Vorstellung	Ohne RB	RB	RB

*PsA* Psoriasisarthritis; *AI* Autoimmunerkrankungen; *AInf* autoinflammatorische Erkrankungen; *RB* Rheumaboard

^a^Acne inversa, CREST (Kalzinose, Raynaud-Syndrom, gestörte Peristaltik im Ösophagus, Sklerodaktylie und Teleangiektasie), Dermatomyositis, Sharp-Syndrom, systemischer Lupus erythematodes, systemische Sklerose, sonstige Kollagenosen

^b^Sonstige Erkrankungen: Sjögren-Syndrom, Morbus Behçet, Pemphigus vulgaris, SAPHO(Synovitis, Akne, Pustulosis, Hyperostosis und Osteitis)-Syndrom

## Ergebnisse

### Zeitspanne von Vorstellung in der Dermatologie bis zur Therapieeinleitung bzw. Therapieoptimierung

**Figure 2:**
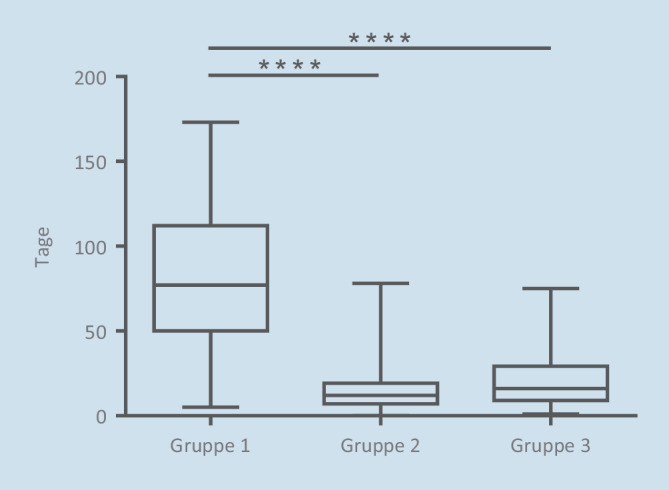

In der ersten Gruppe warteten die Patienten 85 ± 42,24 (Mittelwert ± Standardabweichung) (5 bis 173) Tage auf die konsiliarische Vorstellung in der Rheumatologie. Zwei Patienten stellten sich notfallmäßig in der rheumatologischen Sprechstunde vor. Sie waren in der Dermatologie mit der Diagnose PsA bekannt, wurden aber nicht konsiliarisch angefragt. Diese Patienten wurden in dieser Auswertung nicht mitbeurteilt. In der zweiten Gruppe warteten die Patienten 15 ± 13,09 (0 bis 78) Tage und in der dritten Gruppe 20 ± 16,71 (1 bis 75) Tage, um im RB besprochen zu werden, was in den beiden Gruppen signifikant schnellere Mitbetreuung durch den Rheumatologen brachte (*p* ≤ 0,0001), im Vergleich zu der ersten Gruppe (Abb. 2); 24 Patienten aus der Gruppe 2 und 5 Patienten aus der Gruppe 3 wurden aus dieser Auswertung ausgeschlossen, da sie ursprünglich für die konsiliarische Vorstellung in der rheumatologischen Sprechstunde geplant wurden (während des ersten Beobachtungszeitraums), aber bei fehlenden freien Terminen letztlich im RB besprochen wurden.

### Diagnosesicherung/-bestätigung/-ausschluss nach Mitbetreuung durch die Rheumatologie

**Figure 3:**
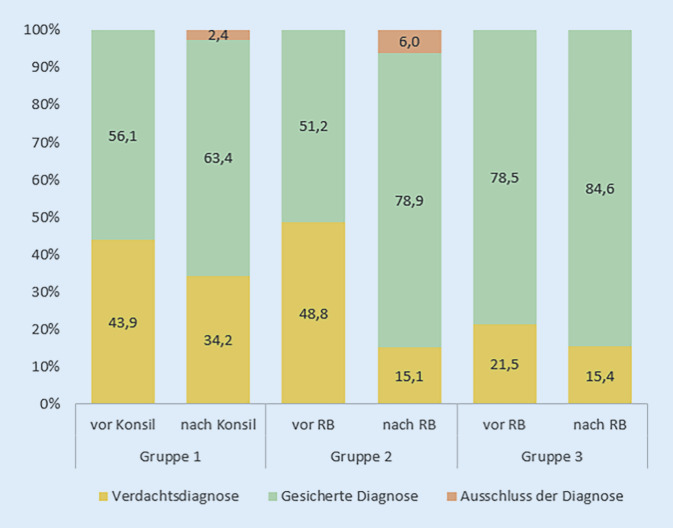

Nach der konsiliarischen Vorstellung in der Rheumatologie wurde in Gruppe 1 bei 26 Patienten (63,4 %) die Diagnose bestätigt oder gesichert, bei 1 Patienten (2,4 %) wurde die Diagnose ausgeschlossen, und bei 14 Patienten (34,2 %) bestand weiterhin der Verdacht einer PsA. Nach der Vorstellung im RB wurde in Gruppe 2 bei 131 Patienten (78,9 %) die Diagnose bestätigt oder gesichert, bei 10 Patienten (6 %) wurde die Diagnose ausgeschlossen, und bei 25 Patienten (15,1 %) bestand weiterhin der Verdacht einer PsA. In Gruppe 3 wurde die Diagnose bei 55 Patienten (84,6 %) bestätigt oder gesichert, und bei 10 Patienten (15,4 %) bestand weiterhin eine Verdachtsdiagnose (Abb. 3).

### Therapieweiterführung/-optimierung nach Mitbetreuung durch die Rheumatologie

**Figure 4:**
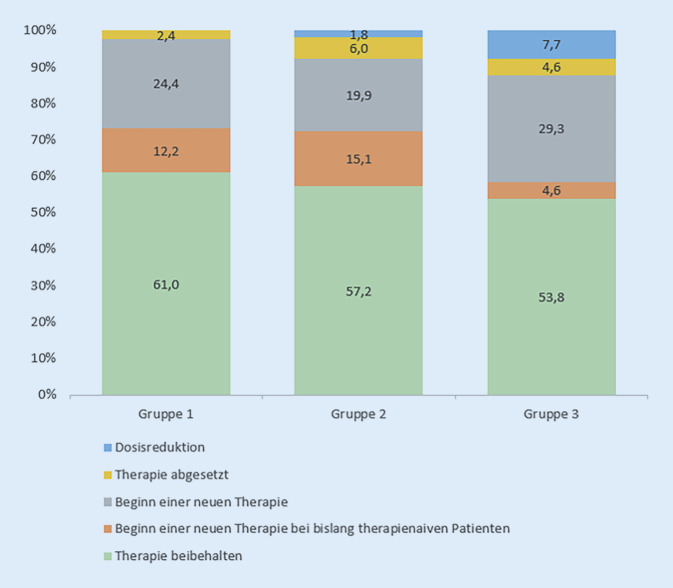

Durch die interdisziplinäre Betreuung der Patienten (konsiliarisch oder im RB) wurden je nach Diagnose, Symptomatik und Ergebnis der Diagnostik sowie Komorbidität in den 3 Gruppen Therapien neu begonnen, weitergeführt, in der Dosis verändert oder abgesetzt. In Gruppe 1 wurde bei 25 Patienten (61 %) die Therapie beibehalten und bei 16 Patienten (39 %) verändert (2,4 % abgesetzt, 24,4 % Beginn einer neuen Therapie, 12,2 % Beginn einer neuen Therapie bei bislang therapienaiven Patienten). In Gruppe 2 wurde bei 95 Patienten (57,2 %) die Therapie beibehalten und bei 71 Patienten (42,8 %) verändert (1,8 % Dosisreduktion, 6,0 % abgesetzt, 19,9 % Beginn einer neuen Therapie, 15,1 % Beginn einer neuen Therapie bei bislang therapienaiven Patienten). In Gruppe 3 wurde bei 35 Patienten (53,8 %) die Therapie beibehalten und bei 30 Patienten (46,2 %) die Therapie verändert (7,7 % Dosisreduktion, 4,6 % abgesetzt, 29,3 % Beginn einer neuen Therapie, 4,6 % Beginn einer neuen Therapie bei bislang therapienaiven Patienten) (Abb. 4).

## Diskussion

Die interdisziplinäre medizinische Patientenversorgung nimmt nicht nur in der Onkologie einen immer größeren Stellenwert ein. In der Universitätsmedizin Mainz findet seit September 2015 eine regelmäßige (zunächst 2‑wöchentliche [09/2015 bis 01/2018]/mittlerweile wöchentliche [02/2018 bis dato]) Vorstellung der Patientenfälle im RB statt. Dadurch werden die Patienten mit fachübergreifenden und komplexen AI/AInf interdisziplinär, individuell und nach den neuesten Erkenntnissen der medizinischen Möglichkeiten betreut. In unserer retrospektiven Auswertung haben wir 3 Patientengruppen mit gesicherter PsA oder dem Verdacht auf eine solche sowie mit anderen AI/AInf untersucht (Tab. 1). In Gruppe 1 warteten die Patienten durchschnittlich 85 Tage auf die konsiliarische Vorstellung in der Rheumatologie. In der Gruppe 2 wurden die Patientenfälle im Durchschnitt nach 15 Tage und in der Gruppe 3 nach 20 Tagen im RB vorgestellt und diskutiert. Wir konnten zeigten, dass die Vorstellung der Patienten bei Rheumatologen im Rahmen des RB signifikant schneller stattgefunden hat als eine konsiliarische persönliche Vorstellung der Patienten. Somit konnte bei diesen Patienten die Diagnose schneller gesichert, bestätigt oder ausgeschlossen werden und die Wartezeit bis zum Beginn einer individuellen Therapie bzw. Anpassung des bestehenden Therapiekonzeptes reduziert werden. Grund dafür könnte die interne Vorgehensweise in der Rheumatologie sein, wie z. B. Terminvergabe bzw. Terminpriorisierung bei der Anforderung eines Konsils vs. einer Vorstellung im RB. Unsere Ergebnisse spiegeln den Alltag wider. Der Personalmangel, der erhöhte Zeitaufwand bei einer konsiliarischen Mitbeurteilung sowie die stets zunehmende Patientenanzahl stellen eine organisatorische und logistische Herausforderung in den Kliniken dar. Auf der anderen Seite kann eine strukturierte Patientenvorstellung mit allen benötigten Unterlagen in einem Board eine schnelle, effiziente und problemorientierte interdisziplinäre Diskussion erlauben.

Die Literaturrecherche zeigte, wie sich auch in anderen multidisziplinären Zentren die Notwendigkeit der fachübergreifenden Behandlung positiv darstellte. Onkologische Boards finden seit Jahren in Kliniken mit verschiedenen Fachrichtungen meist wöchentlich statt [1, 15]. Mehrere Fachdisziplinen erstellen anhand von den Befunden der Patienten, unter Berücksichtigung der Komorbidität und Lebensqualität, optimale Therapiekonzepte und Empfehlungen für den Patienten. Das onkologische Board aus Lublin kam zum Ergebnis, dass die moderne onkologische Therapie und Behandlung nur durch modern ausgestattete interdisziplinäre Zentren ein ausreichend hohes Niveau zeigt. Die optimale Behandlung und Strategie führen sogar zur signifikanten Verbesserung der 5‑Jahres-Überlebensrate [15]. In einer weiteren Studie wurde ein Krankenhaus in Glasgow mit einem multidisziplinären Team untersucht, das Patienten mit inoperablem nichtkleinzelligem Lungenkrebs betreut hat. Es wurde die Therapieänderung durch das multidisziplinäre Team Chemotherapie vs. palliative Therapie analysiert. Das Ergebnis zeigte neben Therapieänderungen durch das multidisziplinäre Team auch eine signifikante Verlängerung des Überlebenszeitraumes [7]. Eine multidisziplinäre Betreuung kann die Diagnosestellung und die Therapie auch bei Patienten mit Psoriasis und PsA erleichtern [18]. Das Massachusetts General Hospital publizierte 2020 die Ergebnisse einer seit 9 Jahren bestehenden Zusammenarbeit zwischen Dermatologen und Rheumatologen. Diese arbeiten in einer kombinierten Klinik multidisziplinär zusammen und führen daraus folgend umfassendere Haut- und Gelenkuntersuchungen durch. Es zeigte sich ein optimiertes Management mit intensiver Diagnostik und Therapie und dadurch resultierender verbesserter Krankheitskontrolle [3].

Die interdisziplinäre medizinische Versorgung von Patienten mit AI/AInf in der Dermatologie und Rheumatologie ist unverzichtbar v. a. aufgrund eines ansteigenden Patientenaufkommens und der Komplexität der Krankheitsbilder mit mittlerweile den verschiedensten Therapieoptionen. Wichtig bei der Versorgung der AI/AInf-Patienten ist die interdisziplinäre und zeitnahe Diagnostik, frühe Diagnose und suffiziente Therapie, um Komorbidität und Symptomprogredienz zu verhindern und dabei ein optimales Therapiemanagement inklusive Monitoring zu erarbeiten. Es ist daher relevant, dass diese Patienten frühestmöglich von Spezialisten behandelt werden und durch die frühzeitige Hemmung der entzündlichen Aktivität die Remission erreichen, gefolgt von Verbesserung der Lebensqualität.

## Fazit für die Praxis

- Vorstellung der Patientenfälle im Reumaboard findet signifikant schneller statt als eine Vorstellung der Patienten bei Rheumatologen mit einem persönlichen Vorstellungstermin.
- Durch die Etablierung eines multidisziplinären Boards zeigt sich eine signifikante Verkürzung der Zeitspanne zwischen Vorstellung und Diagnosesicherung sowie Therapieeinleitung.
- Eine gute Zusammenarbeit zwischen Dermatologen und Rheumatologen bringt nicht nur einen deutlichen Gewinn für die Patientenversorgung bei Patienten mit komplexen Autoimmun- und/oder autoinflammatorischen Erkrankungen, sondern ermöglicht auch ein ungeahntes Weiterbildungspotenzial für die Ärzte.
